# Ten simple rules for using large language models in science, version 1.0

**DOI:** 10.1371/journal.pcbi.1011767

**Published:** 2024-01-31

**Authors:** Gabriel Reuben Smith, Carolina Bello, Lalasia Bialic-Murphy, Emily Clark, Camille S. Delavaux, Camille Fournier de Lauriere, Johan van den Hoogen, Thomas Lauber, Haozhi Ma, Daniel S. Maynard, Matthew Mirman, Lidong Mo, Dominic Rebindaine, Josephine Elena Reek, Leland K. Werden, Zhaofei Wu, Gayoung Yang, Qingzhou Zhao, Constantin M. Zohner, Thomas W. Crowther

**Affiliations:** 1 Department of Environmental Systems Science, Institute of Integrative Biology, ETH Zürich, Zürich, Switzerland; 2 Department of Genetics, Evolution and Environment, University College London, London, United Kingdom; 3 Anarchy, San Francisco, California, United States of America; 4 College of Water Sciences, Beijing Normal University, Beijing, China; 5 Sorbonne Université, Paris, France; 6 College of Resources and Environmental Sciences, Nanjing Agricultural University, Nanjing, Jiangsu, China; Carnegie Mellon University, UNITED STATES

## Introduction

Generative artificial intelligence (AI) tools, including large language models (LLMs), are expected to radically alter the way we live and work, with as many as 300 million jobs at risk [1]. Arguably the most well-known LLM currently is GPT (generative pre-trained transformer), developed by American company OpenAI [2]. Since its release in late 2022, GPT’s chatbot interface, ChatGPT, has exploded in popularity, setting a new record for the fastest growing user base in history [3]. The appeal of GPT and other LLMs stem from their ability to effectively carry out multistep tasks and provide clear, human-like responses to complicated queries and prompts (Box 1). Unsurprisingly, this capacity is catching the eye of scientists [4].

**Box 1—An LLM suggests 10 simple rules governing its own usage in science**

Here, we ask ChatGPT to generate a list of 10 simple rules for using LLMs in science in order to illustrate its ability to provide detailed, human-like responses to short prompts.

**Q1**: Can you provide me with a list of ten simple rules for using large language models in science?

**R1 (ChatGPT 3.5)**: Certainly! Here are ten simple rules for using large language models in science:

1. Understand the limitations: Recognize that large language models like GPT-3 are powerful but not infallible. They may produce accurate-sounding information, but they can also generate false or biased content. Exercise caution and critical thinking.
2. Validate and verify: Always cross-reference information generated by language models with reliable sources. Verify the accuracy of claims, check for consistency, and corroborate the findings through empirical evidence or expert opinions.
3. Train with caution: When fine-tuning a language model for a specific scientific task, carefully design and curate the training data. Ensure the data is representative, diverse, and free from biases that may influence the model’s outputs.
4. Provide context and instructions: Clearly specify the intended context and constraints when using a large language model. This helps narrow down the results and ensures the model generates more relevant and accurate responses.
5. Understand the biases: Large language models learn from the data they are trained on, which can contain societal biases. Be aware of these biases and take steps to mitigate them by using diverse training data and evaluating the outputs for fairness.
6. Collaborate and share knowledge: Engage in open dialogue with other researchers and scientists working with language models. Share experiences, discuss challenges, and collectively work towards best practices for using these models in scientific research.
7. Maintain transparency: When presenting work that incorporates the use of large language models, disclose the methodology, tools, and limitations. Be transparent about the role of the language model in the research process and acknowledge its potential impact on the results.
8. Ethical considerations: Consider the ethical implications of using language models in science. Be mindful of privacy concerns, consent, and potential misuse of generated content. Respect ethical guidelines and seek approval when working with sensitive or personal data.
9. Avoid overreliance: Large language models can be a valuable tool, but they should not replace critical thinking, domain expertise, or rigorous scientific methods. Use them as a supportive tool alongside other research methodologies.
10. Contribute to responsible AI development: Actively participate in efforts to improve large language models and promote responsible AI practices. Provide feedback to developers, collaborate on research, and advocate for ethical and inclusive use of these models in science and society.

Remember, these rules provide general guidance, and it’s important to adapt them to specific scientific disciplines and contexts.

Indeed, there is increasing interest in using GPT and other LLMs to accelerate scientific progress for the benefit of humankind [5]. However, specific challenges concerning possible misuse of LLMs in science are arising [6] in tandem with broader concerns about potential societal disruption and ethical risks [7,8]. As such, there is an urgent need for the scientific community to establish general guiding principles for the appropriate use of LLMs and other generative AI tools to maximise benefit and minimise harm [9,10].

Here, we propose a set of 10 simple rules for using LLMs in science, drawn from our own experimentation as cautiously optimistic environmental scientists curious about novel tools to streamline research. We note that the list is grounded in our expertise as scientists and experience as end-users of LLMs (GPT specifically), not as AI developers. We also note that we do not here address other sorts of generative AI, which could also be increasingly used for scientific research in the future.

We suggest safeguards against 5 areas of concern to be wary of (**Rules 1 to 5**), complemented by suggestions for areas where LLMs have potential to support scientific research if sufficient care is taken to avoid issues (**Rules 6 to 10**). Since LLMs are predictive language models, our use suggestions focus on language-centric aspects of scientific research, such as computer coding, writing, and publishing.

As developments in this field are rapid and outcomes often unpredictable [11], we envision that these guidelines can provide a starting point, not an end point; they will likely need to be revisited and adapted as circumstances change. We envision, additionally, that our list may also provide a basis for better standardised reporting and documentation (S1 Appendix) usable across journals, allowing researchers who are submitting manuscripts to document their use(s) of LLMs and affirm that they have appropriately considered potential problem areas.

### Safeguards

#### 1. Follow the rules of the target journal

It’s essential to consult and follow an up-to-date version of the rules for the target journal prior to using an LLM for research. As these tools gain in popularity, journals are likely to provide explicit guidelines on what they consider to be acceptable or unacceptable uses in submitted manuscripts. Several journals have already issued statements on this topic [12], but these guidelines may be updated and changed as scientists increasingly experiment with LLMs and discover new uses. In fact, we anticipate that a key element of scientific ethics may soon concern proper disclosure of generative AI usage for research.

Importantly, different journals might adopt substantially different policies. If this occurs, work developed with one journal in mind could be fundamentally inadmissible in another even if the topic and novelty would otherwise be well-matched. This problem could potentially be mitigated by alignment along a standardised framework for reporting of generative AI use in science. We include an example document of this sort in the appendix of this paper (S1 Appendix), specifically for LLMs and based upon the rules given here. This document should be adapted and modified as new or use-specific challenges arise. If there is uncertainty concerning a given use, we encourage authors to discuss with editors and make use of the acknowledgements section of their papers to disclose their use of generative AI with sufficient detail.

#### 2. Outline relevant risks before LLM use

Because LLM use can lead not only to benefit but also to harm [13], researchers who would like to use one for their project should also first outline relevant risks [14]. Based on an assessment of the risks posed by a particular use case, an approach can be decided upon that maximises benefit and minimises potential harm.

For some risks, mitigation may be straightforward. For example, erroneous LLM-generated content can be caught and corrected by thoroughly and critically proofreading (**Rule 5**). However, other well-documented LLM risks are too complex to be amenable to complete mitigation by a single researcher or team. In these cases, a researcher can instead qualitatively evaluate the extent to which their project might exacerbate the problem and make modifications to limit the specific negative impact of their work.

For example, if LLMs prove to be a boon to scientific research, variation in LLM accessibility and user-skill risks contributing to an unequal playing field for scientists competing for funding and employment. For this problem, a partial solution may take the form of researchers making LLM prompts they have used for their research freely available in the spirit of open science, so that others may learn from them. A second concern revolves around biases in a model’s underlying training data, which could cause its output to not only reinforce harmful societal prejudices [13,14] but also hamper scientific creativity by hewing closely to existing scientific narratives without emphasising knowledge gaps [15,16]. This problem may be addressed in part by checking LLM-generated content specifically for evidence of bias and using LLMs only in later stages of scientific work, after creative inquiry and hypothesis testing have already occurred.

#### 3. Avoid plagiarism

Because generative AI presents new challenges for legal frameworks surrounding copyright and intellectual property, researchers must proactively ensure that their LLM use is not considered plagiarism within its relevant context. Indeed, beyond transparently unethical uses of LLMs, for example, to defeat plagiarism-detection software [17], subtle issues surrounding plagiarism can also arise in everyday use of LLMs for science.

As long as an LLM is not considered a legal person, unattributed use of the text they produce would not seem to be plagiarism under most current legal definitions. However, it may be inadmissible for other reasons—for example, directly incorporating text from GPT into a manuscript currently violates some journal rules [12], rendering this an unacceptable usage (**Rule 1**). Nevertheless, debate continues about how precisely guidelines and concepts of academic integrity ought to evolve in response to the increasing availability of generative AI [18,19], with no clear settled consensus yet. We anticipate that norms surrounding incorporation and declaration of LLM-generated text (after fact-checking) in scientific literature may change considerably in the near future. Currently, researchers might best conceptualise LLM-produced text as a third-party source, which can inform but not replace their own writing.

#### 4. Respect confidentiality

Unlike use of standard analytical tools, sharing confidential data or information with an LLM represents a potential breach of contract and must be avoided unless explicit permission is obtained. This is important to note because researchers wishing to debug computer code (**Rule 6**), summarise content (**Rule 7**), or improve manuscript writing (**Rule 10**) might wish to share code, data, or writing with an LLM.

Though tempting, doing so presents a major potential problem because it remains unclear whether GPT in particular might “remember” this information and then share it with third parties who ask similar questions. In fact, OpenAI states that conversations that GPT (in the context of OpenAI consumer services) has are indeed used for further training of the model [20]. Scientists who work with sensitive, confidential data, for example medical records, must keep this in mind. Similar caution may be called for regarding preliminary scientific findings that are not yet ready for publication. We suggest that until there is sufficient clarity on this topic, data of this sort should not be transmitted to GPT, even if data confidentiality agreements (which may have been drafted before the advent of widespread LLM availability) do not explicitly rule out such an activity.

#### 5. Verify the truthfulness of content generated by an LLM

Content generated by an LLM must always be double-checked by a subject matter expert for factual accuracy. LLMs are well known to “hallucinate”—in other words, to give seemingly correct but false answers—particularly in response to complex questions (Box 2) [21]. Problematically, the model’s eloquence can cause users to overlook false statements or references. This issue is an example of the “halo effect,” a well-known cognitive bias where an individual’s specific positive traits (e.g., eloquence) are mistakenly interpreted as evidence of a general “goodness” that extends beyond these specific traits [22]. In the case of an LLM, the quality of the presentation may be unconsciously assumed to match the veracity of the content.

Safeguarding against hallucinations may be easy or difficult, depending on the nature of the task that the LLM has been asked to perform. For example, a citation can be quickly cross-checked for accuracy and code can simply be executed to test whether it does what it is supposed to. But if one asks an LLM to summarise an article or transcribe a meeting (as in **Rule 7**), checking for accuracy might be much more challenging and time-consuming. The difficulty of checking in these cases might tempt users to trust responses uncritically—a tendency against which we must be on guard.

**Box 2—Examples of LLM hallucination**

Here, we request factual, referenced information from ChatGPT. In response, we receive apparently convincing answers which actually contain subtle, potentially misleading inaccuracies.

**Q1**: Provide a referenced estimate of the amount of vegetation biomass on planet Earth.

**R1 (ChatGPT3.5):** As of my knowledge cutoff in September 2021, estimating the exact amount of vegetation biomass on planet Earth is challenging due to the vastness and complexity of ecosystems. However, scientists have made various estimations using different methods and data sources. It’s important to note that these estimates can vary depending on the specific criteria and assumptions used in the calculations.

One study published in the journal Nature in 2020 provides an estimate of global vegetation biomass based on satellite observations and ground measurements. The research suggests that the Earth’s total vegetation biomass is approximately 450 billion metric tons (gigatons). This includes all the vegetation on land, including forests, grasslands, shrublands, and other types of vegetation.

Here is the reference for the study:

Zhu, Z., et al. (2020). Greening of the Earth and its drivers. Nature, 597(7871), 405-409. doi: 10.1038/s41586-020-2474-6.

Please keep in mind that new studies and advancements in remote sensing technologies may have provided more accurate estimates since my last update. It is advisable to consult recent scientific literature for the most up-to-date information on the subject.

**Inaccuracies:** This is a good example of a seemingly correct answer with some critical mistakes. The paper “Greening of the Earth and its drivers” was indeed published by Zhu and colleagues [23], but in *Nature Climate Change*, 2016 (doi: 10.1038/nclimate3004), not in *Nature*. Moreover, in Zhu and colleagues (2016), no estimate was provided of Earth’s total vegetation biomass. The estimate of 450 Gt C can instead be traced to Bar-On and colleagues (2018) [24].

**Q2:** What are the geospatial patterns of soil nematodes at a global scale? Provide references to the statements provided.

**R2 (ChatGPT4):** As of my last update in September 2021, the most comprehensive study on the global distribution of soil nematodes was published in 2019 in the journal "Nature". The research was conducted by Van den Hoogen et al., who assembled a global soil nematode dataset covering all continents and biomes.

Here are some of the key findings from the study:

Soil nematodes are ubiquitous and are found in every type of ecosystem, from the tropics to the Arctic and Antarctic, and from lowland areas to high mountain ranges.

Nematode density, or the number of nematodes per unit of soil, tends to decrease with increasing latitude. This means that nematode densities are generally higher in the tropics than in temperate or polar regions.

The highest nematode densities were found in grasslands and forests, and the lowest densities were found in deserts and tundra.

Nematode communities vary greatly in their composition depending on the ecosystem. For example, bacterial-feeding nematodes are more common in ecosystems with high organic matter content, while fungal-feeding nematodes are more common in ecosystems with low organic matter content.

The total global soil nematode population was estimated to be around 0.3 x 10^20 (or 30 quintillion) individuals, making them the most abundant group of animals on Earth.

Reference:

Van den Hoogen, J., Geisen, S., Routh, D., Ferris, H., Traunspurger, W., Wardle, D. A., & & Crowther, T. W. (2019). Soil nematode abundance and functional group composition at a global scale. Nature, 572(7768), 194-198. doi: 10.1038/s41586-019-1418-6

**Inaccuracies:** Again, the answer provided is seemingly correct but contains errors. In the referenced paper [25], the estimated number of soil nematodes is actually 4.4 × 10^20, with a total biomass of 0.3 Gt. Additionally, the latitudinal patterns described in the paper are the opposite from ChatGPT’s answer. Finally, the paper contains no statements supporting a link between bacterial-feeding or fungal-feeding nematodes and organic matter content.

### Use suggestions

#### 6. Use an LLM to perform a more inclusive data search

An LLM can assist researchers with data gathering for scientific meta-analyses or syntheses by collating “grey literature” resources—reports issued by governmental and nongovernmental organisations rather than scientific journals. This is helpful because while there are established methods for systematically searching the scientific literature using databases such as Web of Science [26], these approaches may neglect grey literature [27]. This oversight is particularly problematic in applied fields such as conservation and ecological restoration, where the insights of on-the-ground practitioners are of central importance.

Researchers can use GPT and other LLMs to collate and synthesise findings from grey literature (Box 3), which allows for easier incorporation into systematic reviews and facilitates knowledge sharing across organisations [28]. However, unless the LLM has internet access (as with some GPT plugins or Microsoft’s NewBing), the ability of the model to excel at this task may be limited by how comprehensive and recent its training corpus is. In the example below (Box 3), ChatGPT provides a helpful overview of organisations with relevant information but some finer-scale details (e.g., ChatGPT’s estimation of website contents) are erroneous, either due to model hallucination (**Rule 5**) or, potentially, changes occurring after the training data snapshot was taken.

**Box 3—Performing an LLM-assisted grey literature search**

Here, we use ChatGPT to generate a preliminary list of organisations that work in some aspect of forest restoration and which may have published reports online detailing the impact of their work.

**Q1:** Please generate a list of forest restoration organizations that publish data on the biodiversity and carbon outcomes of their work.

**R1:** Certainly! Here is a list of forest restoration organizations that publish data on the biodiversity and carbon outcomes of their work:

1. The Nature Conservancy: The Nature Conservancy is a global environmental organization that works to protect and restore critical habitats, including forests. They publish regular reports on their conservation efforts, including data on biodiversity and carbon sequestration.

2. Trees for the Future: Trees for the Future is a non-profit organization that works with local communities to plant trees and restore degraded lands. They publish annual reports that detail the impact of their work, including data on biodiversity and carbon sequestration.

3. Rainforest Alliance: The Rainforest Alliance is an international organization that works to conserve biodiversity and promote sustainable livelihoods through responsible land use practices. They publish regular reports on their forest conservation efforts, including data on carbon sequestration and biodiversity.

4. World Wildlife Fund: The World Wildlife Fund is a global conservation organization that works to protect and restore critical habitats, including forests. They publish regular reports on their conservation efforts, including data on biodiversity and carbon sequestration.

5. Forest Stewardship Council: The Forest Stewardship Council is an international organization that promotes responsible forest management practices. They publish regular reports on their certification process.

**Q2:** Have these organizations published reports on their work online?

**R2:** Yes, these organizations typically have published reports on their work available online. While I don’t have real-time access to the internet, I can provide you with general information about where to find these reports. You can visit the websites of these organizations and look for sections such as “Publications,” “Reports,” or “Impact” to find detailed information about their forest restoration projects, including data on biodiversity and carbon outcomes.

For example:

1. The Nature Conservancy: Visit their website at nature.org and explore their “Our Work” or “Publications” sections.

2. Trees for the Future: Visit their website at trees.org and look for sections like “Impact” or “Resources” for access to their reports.

3. Rainforest Alliance: Visit their website at rainforest-alliance.org and search for sections such as “Our Work” or “Publications” to find their reports.

4. World Wildlife Fund: Visit their website at wwf.org and explore their “Conservation” or “Publications” sections for access to their reports.

5. Forest Stewardship Council: Visit their website at fsc.org and look for sections like “Resources” or “Publications” for their reports on responsible forest management.

Please note that the availability and specific locations of these reports may vary over time, so it’s best to explore the respective websites or use their search functions to locate the most up-to-date information.

**Q3:** Please turn this into a table that facilitates finding and extracting data from the reports for these organizations, and 5 other relevant organizations.

**R3:** Certainly! Here is a table that includes the organizations mentioned earlier, as well as five additional forest restoration organizations, along with relevant information that can facilitate finding and extracting data from their reports:

[Table not shown for brevity, see Q4 for follow up that adds column on geographical scope]

Note that this table is not exhaustive and there may be additional relevant sections and types of reports available on each organization’s website. However, this should provide a good starting point for finding and extracting data from their reports.

**Q4:** Please add a column to this table that describes the geographic scope of each organization.

**R4:** Certainly! Here is an updated table that includes the organizations mentioned earlier, along with five additional forest restoration organizations, relevant sections on their website, types of reports they publish, and the geographic scope of their work:

**Table:** 

**Organization Name**	**Website**	**Relevant Sections**	**Types of Reports**	**Geographical Scope**
The Nature Conservancy	https://www.nature.org/	Our Work, Publications	Annual reports, Impact reports, Biodiversity and Carbon reports	Global
Trees for the Future	https://trees.org/	Impact, Resources	Annual reports, Impact reports, Biodiversity and Carbon reports	Africa, Asia, Latin America, and the Caribbean
Rainforest Alliance	https://www.rainforest-alliance.org/	Our Work, Publications	Annual reports, Impact reports, Biodiversity and Carbon reports	Global
World Wildlife Fund	https://www.worldwildlife.org/	Conservation, Publications	Annual reports, Impact reports, Biodiversity and Carbon reports	Global
Forest Stewardship Council	https://fsc.org/	Resources, Publications	Certification reports, Biodiversity and Carbon reports	Global
Eden Reforestation Projects	https://edenprojects.org/	Impact, Stories	Annual reports, Impact reports, Biodiversity and Carbon reports	Africa, Asia, Haiti, Madagascar, and Central America
The Arbor Day Foundation	https://www.arborday.org/	Programs, Reports	Annual reports, Impact reports, Biodiversity and Carbon reports	United States and internationally
American Forests	https://www.americanforests.org/	Our Work, Publications	Annual reports, Impact reports, Biodiversity and Carbon reports	United States and internationally
Trees, Water & People	https://www.treeswaterpeople.org/	Our Programs, Reports	Annual reports, Impact reports, Biodiversity and Carbon reports	Latin America and the United States
Conservation International	https://www.conservation.org/	Our Work, Publications	Annual reports, Impact reports, Biodiversity and Carbon reports	Global


Note that the geographic scope of each organization’s work may vary over time, and this information is accurate as of the knowledge cutoff date for my training.

All organisations identified were relevant and many publish data or summaries of their impacts in annual reports somewhere on their websites. An extended list of additional organisations was provided with 1 additional prompt, and it is easy to export the list in a standard format (e.g.,.csv). However, the list does contain several inaccuracies (see **Rule 5**). For example, the “Relevant Sections” column often listed website sections that did not actually exist.

#### 7. Use an LLM to summarise content

To help researchers make more efficient use of their time, an LLM can quickly generate short summaries based on lengthy input content. This is helpful because as a scientist, it’s essential to regularly read new literature, but balancing this task with the duties of our own research, teaching, and supervision can be challenging. Yet, researchers who fail to strike a good balance and don’t frequently investigate new literature can miss out on findings that might helpfully inform and shape their own research programs, or which would be relevant to classes that they are teaching.

Currently, several third-party plugins for GPT (e.g., ChatPDF.com, UseChatGPT.AI, GPT Academic, SciSpace copilot) can be used to generate summaries based on open-access scientific literature, complete with main points, findings, and contributions. Microsoft’s NewBing search engine, which incorporates GPT-4, can also be used for this purpose. With tailored structural prompts (Box 4), a researcher can specify the length, style, and format they prefer for a summary. ChatGPT can also respond to follow-up questions requesting more details, if needed, producing a kind of interactive abstract. Yet, just as with standard abstracts, if a work appears to be of interest, appropriately contextualising and fully understanding the main findings requires that a researcher read the full paper.

**Box 4—Using GPT to summarise scientific literature**

Here, we use GPT-4 (via NewBing) to summarise a recently published open-access scientific paper.

**Q1:** I want you to act as a scientific paper summarizer. I will give you the URL of a scientific article. I want you to reply with a bold title for each section including the general information of this paper, the background of this research, the addressed question and hypothesis, the key findings, and major contributions. The summary of each section should be logical, concise, clear, and informative. Make sure the summary includes most of the key information and can grip the audience. The URL is: https://www.nature.com/articles/s41467-022-30888-2.

**R1 (GPT-4 *via* NewBing): General information:** The article is titled “Global relationships in tree functional traits” and it was published in Nature Communications on 08 June 2022. The authors are Daniel S. Maynard, Lalasia Bialic-Murphy, Constantin M. Zohner, Colin Averill, Johan van den Hoogen, Haozhi Ma, Lidong Mo, Gabriel Reuben Smith, Alicia T. R. Acosta, Isabelle Aubin, Erika Berenguer, Coline C. F. Boonman, Jane A. Catford, Bruno E. L. Cerabolini, Arildo S. Dias, Andrés González-Melo, Peter Hietz, Christopher H. Lusk, Akira S. Mori, Ülo Niinemets, Valério D. Pillar, Bruno X. Pinho, Julieta A. Rosell, Frank M. Schurr and Thomas W. Crowther1.

**Background:** Trees are subject to unique physiological, mechanical and ecological pressures due to their massive energetic investments in woody support structures. Functional traits are the physiological and morphological features that determine how trees interact with, influence and respond to their environment. Understanding the patterns and trade-offs in tree functional traits is challenging because of the diversity and complexity of tree form and function.

**Question and hypothesis:** The authors aim to quantify the multidimensional relationships in tree trait expression across 18 functional traits encompassing leaf, seed, bark, wood, crown and root characteristics. They hypothesize that these traits reflect two main axes: one related to leaf economics (i.e., the trade-off between resource acquisition and conservation) and the other related to tree size and competition for light (i.e., the trade-off between growth and survival). They also expect that these axes show environmental convergence across temperature, moisture and elevation gradients.

**Key findings:** The authors find that nearly half of trait variation is captured by the two hypothesized axes1. They also find that these axes exhibit correlated responses to environmental factors1. Moreover, they identify eight distinct clusters of traits that capture the full dimensionality of trait space. These clusters reflect different aspects of tree form and function such as drought tolerance, shade tolerance, dispersal ability and nutrient cycling.

**Major contributions:** The authors identify a core set of traits needed to quantify global patterns in functional biodiversity. They also contribute to the fundamental understanding of the functioning of forests worldwide by revealing how tree traits shape their water, nutrient and light economies1. They suggest that their trait framework can be used to improve forest management and conservation strategies.

An LLM can even be used to produce summaries of internal documents, for example meeting minutes, if meeting participants give their consent and no confidential information was shared (**Rule 4**). To facilitate this process, speech-to-text transcription software can first be used to produce a full written record of a meeting. Then, this transcribed meeting can be shared with the LLM, which can be tasked with producing a summary. For this use, the adequacy of the summary should of course be verified by the meeting participants to ensure that important points have not been left out nor misrepresented.

#### 8. Use an LLM to refine written English in formal and informal communication

An LLM can facilitate clearer and more idiomatic usage of English, the *lingua franca* of the international scientific community. This can be especially helpful for researchers who speak English as a second language, who may face unique obstacles in the English-dominated environment of global science.

Unfortunately, peer review tends to disadvantage authors from the developing world and non-English speaking countries [29] and researchers who speak English as a second language sometimes receive rude, discouraging remarks from referees [30]. Coupled with journal adoption of double-blind reviewing practices [31], an LLM can help to address this problem by copyediting manuscripts and other communication to ensure that English is used idiomatically [32], reducing opportunities for bias from referees. Importantly, before using an LLM in this way journal guidelines must be consulted (**Rule 1**). If such a use is not explicitly permitted under journal policy, we suggest the model’s role as manuscript copyeditor should be directly communicated to journal editors.

An LLM’s ability to proofread and copyedit can also be helpfully extended to informal communication as well. For example, an LLM can also be used to verify that e-mails and presentations written in English capture the appropriate tone, which can be a challenge even for researchers whose first language is English. Skilfully handling informal communication is important for researchers who seek to establish collaborative networks with peers they haven’t yet met in person (e.g., [33]). Since collaboration supports both basic scientific progress and scientific career development [34], minimising the barriers posed by language in this sphere promises significant benefit.

#### 9. Use an LLM to improve scientific coding

An LLM can help scientists to write computer code more quickly and efficiently [35], as LLMs excel not only in natural language processing but also in programming tasks across diverse programming languages. Models can be used to generate initial code snippets or to improve the structure, efficiency, and readability of existing code. This can be done, for example, by (i) arranging code into logical sections like functions, classes and modules; (ii) refactoring code by suggesting better naming conventions, eliminating code duplication, and improving overall code readability; (iii) suggesting alternative algorithms or data structures that may improve efficiency and performance of the code; or (iv) writing clearer code annotations.

An LLM can also aid in debugging and error detection (Box 5). Yet, suggestions from LLMs may not necessarily provide the most efficient code or follow established best practices in the field; depending on how recent the training data are, deprecated functions and packages may also be used. In all cases, it’s crucial to verify the functionality of any provided code to ensure that it aligns with the user’s specific needs.

**Box 5—Using GPT to debug computer code**

Here, the user provides ChatGPT with Google Earth Engine code (rendered as […] in the example) that is meant to perform a set of statistical aggregation and spatial calculations within ecoregions [36] but is instead returning an error. ChatGPT identifies the cause of the error and suggests solutions. The initial suggested code returns a new error, but with further prompting ChatGPT successfully corrects the error to produce functional code.

**Q1**: […] Here is my code in Google Earth Engine. I get this message error: Too many concurrent aggregations. Can you help me to find a solution?

**R1**: The error “Too many concurrent aggregations” occurs when you are trying to perform too many reduceRegion or other aggregation operations simultaneously. In your code, you are using both reduceRegion and map functions over a large list of ecoIds. The reduceRegion is applied inside the map function, which may cause too many concurrent aggregation operations to run.

To fix this issue, you can try the following approaches:

1. Split your ecoIds list into smaller chunks and process each chunk separately, then combine the results. You can use the ee.List.slice() function to achieve this.

2. Limit the number of ecoIds you process at once. You can experiment with different numbers of ecoIds to find the optimal number that doesn’t cause the error.

3. You can try to use other Earth Engine functions to perform the operations you need, such as reducing the number of aggregations by using simpler reducers or using other functions that don’t involve aggregation.

Here is an example of how to split the ecoIds list into smaller chunks and process them separately:

// Define a function to process a chunk of ecoIds

function processEcoIdsChunk(chunk) {

var chunkImageCollection = ee.ImageCollection(chunk.map(function(id) {

 // Your existing code for processing each ecoId

}));

return chunkImageCollection.mosaic();

}

// Split the ecoIds list into smaller chunks

var chunkSize = 10; // Adjust this value to find the optimal chunk size

var numChunks = ecoIds.length().divide(chunkSize).ceil();

var ecoIdsChunks = ee.List.sequence(0, numChunks.subtract(1)).map(function(chunkIndex) {

var startIndex = ee.Number(chunkIndex).multiply(chunkSize);

var endIndex = startIndex.add(chunkSize).min(ecoIds.length());

return ecoIds.slice(startIndex, endIndex);

});

// Process each chunk and combine the results

var processedChunks = ecoIdsChunks.map(processEcoIdsChunk);

var imageWithPotValues = ee.ImageCollection(processedChunks).mosaic();

// Add the layer to the map and print results

print(imageWithPotValues);

Map.addLayer(imageWithPotValues);

This code will divide the ecoIds list into smaller chunks and process them one by one, potentially reducing the number of concurrent aggregations and avoiding the error. You may need to experiment with the chunkSize value to find the optimal size that doesn’t cause the error.

**Q2**: “Line 20: chunk.map is not a function” I get this error when running the code. Is this because I didn’t implement the function the right way?

**R2**: My apologies for the confusion. The error is occurring because the ‘chunk’ variable in the ‘processEcoIdsChunk’ function is of type ‘ee.List’, but the ‘map()’ function is being called on it as if it were a JavaScript array. To fix this, you need to cast ‘chunk’ to an ‘ee.List’ before calling the ‘map()’ function on it. Here’s how to do that:

Replace this line:

var chunkImageCollection = ee.ImageCollection(chunk.map(function(id) {

With this line:

var chunkImageCollection = ee.ImageCollection(ee.List(chunk).map(function(id) {

Now, the ‘chunk’ variable is cast to an ‘ee.List’, allowing you to call the ‘map()’ function without issues. This should resolve the error, and your code should work as expected.

An LLM can also facilitate code translation from one programming language to another, which can prove invaluable when packages or libraries needed for a specific analysis are only available in an unfamiliar language. In addition to translating between different programming languages, LLMs can also translate computer code into natural language. In other words, they can explain to a user in intuitive terms what each section of code does and generate documentation directly from the code itself. This can be invaluable when working with sporadically documented code.

Importantly, the effective use of LLMs heavily depends on the user’s ability to craft precise, well-defined prompts that clearly outline the task at hand. In the context of scientific coding, this means the user must already have a strong understanding of the coding language in question as well as a good conceptual comprehension of the task. As such, the current generation of LLMs do not represent an alternative to developing this fundamental capacity, but rather a way to quickly address challenges that arise in coding.

#### 10. Use an LLM to jump-start your scientific writing process

Researchers can overcome writer’s block and “blank page anxiety” by asking an LLM to produce an outline for their scientific paper (Box 1; Box 6). This can be helpful because writing is a major hurdle for many researchers [37], with some of the biggest obstacles posing a challenge at the initial outset of a project’s writing phase [38]. In addition to providing a starting point, an LLM can help to organise subsections, offer relevant examples, and fine-tune the overall flow of the manuscript. In this way, an LLM can act for researchers as a fast and inexpensive proofreader and copyeditor.

**Box 6—Using GPT to produce a tailored paper outline**

**Q1:** Give me a 4-heading structure for a research article. Topic: Climate change effects on biodiversity in the tropics Context: Ecology Language: Academic Tone: Formal.

**R1:** I. Introduction

A. Background on climate change and its global impact

B. Importance of studying climate change effects on biodiversity in the tropics

C. Research question and objectives

II. Literature Review

A. Overview of climate change impacts on biodiversity worldwide

B. Key studies and findings on climate change effects on biodiversity in the tropics

C. Knowledge gaps and research limitations

III. Methodology

A. Study area and data collection

B. Variables and measurements

C. Statistical or analytical methods used

IV. Results and Discussion

A. Presentation of findings related to climate change effects on biodiversity in the tropics

B. Discussion of the results in the context of existing literature

C. Implications and potential future directions

V. Conclusion

A. Summary of the main findings

B. Significance of the research and its contribution to the field

C. Recommendations for future research and policy implications

Importantly, however, an LLM cannot be assumed to provide reliable factual information (see **Rule 5**). Thus, just as with suggestions from a proofreader who may not have subject expertise, erroneous statements can inadvertently appear (admittedly perhaps for different reasons). Additionally, while an LLM can propose a structure for a researcher’s manuscript, there is no guarantee that this structure is the best choice for the author’s purposes. Authors must therefore still carefully evaluate and revise LLM suggestions using their own expertise, while ensuring that they do not directly incorporate LLM-generated text into their manuscript if doing so would contravene journal rules or constitute plagiarism (**Rules 1 and 4**).

## Conclusions

In addition to transforming the world economy [1], generative AI tools like LLMs will likely transform the way we do science [5]. Alongside great potential benefits stand great potential dangers, and within both of these categories are certain to be uses of generative AI that we (and others) have not anticipated. Indeed, as we explore these new tools, we must not lose sight of the unresolved dilemmas that still surround generative AI technology [7,16]. Some of the challenges relevant for research include the use of unspecified, potentially biased training datasets for commercial models [16], an implicit attachment to existing scientific paradigms [15], and a sizeable carbon footprint [39]. Issues such as these are sufficiently complex so as to preclude simple, actionable solutions, such as those we can offer here.

Nevertheless, we suggest that reduction of potential harm in LLM use for science may already be supported by following **Rules 1 to 5**. With these caveats firmly in mind, a charitable researcher may conceive of GPT and other LLMs as a research assistant, copyeditor, or consultant (**Rules 6 to 10**) who is imperfect and does not possess subject knowledge, thus sometimes making erroneous suggestions or statements.

Before beginning, researchers must ensure that their planned LLM use complies with journal guidelines (**Rule 1**) and does not appear to pose substantial, unmitigable risk (**Rule 2**). To avoid plagiarism (**Rule 3**), LLM-generated content should not be used without appropriate attribution, and at no point should confidential information be shared in the course of model usage (**Rule 4**). Finally, due to the possibility of hallucination, all LLM-generated content must be fact checked (**Rule 5**). If these guidelines are respected and regularly revised as circumstances develop, we believe that generative AI tools like LLMs stand to significantly accelerate scientific progress for the benefit of humankind.

## Supporting information

S1 Appendix An example reporting document for large language model use in science. (PDF)
