# Supplementary material for: Ten simple rules for using large language models in science, version 1.0
Source: PLoS Comput Biol. 2024 Jan 31;20(1):e1011767. doi: 10.1371/journal.pcbi.1011767 (PMC10829980; doi:10.1371/journal.pcbi.1011767)
Supplement: S1 Appendix — (PDF) [file pcbi.1011767.s001.pdf]

Appendix 1:

An example reporting document for large language model use in science
from
Ten simple rules for using large language models in science, version 1.0
by Smith et al.

1. Please indicate how large language models (LLM; e.g. OpenAI GPT, Google Bard) were used in the preparation of this manuscript.

N/A: An LLM was not used for this research	☐
Data gathering	☐
Summarising content in the course of literature review	☐
Writing or debugging computer code	☐
Producing an outline or otherwise contributing to a preliminary manuscript draft	☐
Copyediting	☐
Other (please discuss with the journal editor)	☐

2. If you checked any box above besides “N/A: A large language model was not used for this research”, please affirm the following:

I confirm that LLM usage for this research complies with all journal guidelines.	☐
I have outlined risks relevant to LLM use for this project and taken mitigation measures if necessary.	☐
I have ensured that LLM use in this project complies with journal-appropriate plagiarism rules.	☐
In the course of LLM usage, I have ensured that all data confidentiality rules are respected.	☐
I have carefully fact-checked all LLM-generated content employed upstream of final draft writing.	☐

I confirm that I have truthfully answered all questions and have discussed any additional uses with the journal editor.

____

Name, signature *Date, location*

** Note that as of July 2023, incorporation of LLM-generated writing directly into manuscripts is explicitly forbidden by many journal guidelines and is thus not permissible. Should these rules change, we suggest that the presence of LLM-generated content in the manuscript should be declared in response to* ***1.*** *and that the authors must themselves take full responsibility for ensuring the veracity of this content in response to* ***2.***
